# Photoactivated Rose Bengal Triggers Phospholipid Hydroperoxidation and Late Apoptosis in Colorectal Cancer Cells

**DOI:** 10.1021/acs.langmuir.4c05013

**Published:** 2025-03-06

**Authors:** André Satoshi Ferreira, Alexandre Mendes de Almeida Junior, Mirella Boaro Kobal, Lucas Gontijo Moreira, Sabrina Aléssio Camacho, Karina Alves de Toledo, Osvaldo N. Oliveira Jr, Christine E. DeWolf, Pedro Henrique Benites Aoki

**Affiliations:** † São Paulo State University (UNESP), School of Sciences, Humanities and Languages, Assis, SP 19806-900, Brazil; ‡ University of Sao Paulo (USP), São Carlos Institute of Physics, São Carlos, SP 13566-590, Brazil; § Concordia University, Department of Chemistry and Biochemistry and Centre for NanoScience Research, Montreal, QC H4B 1R6, Canada

## Abstract

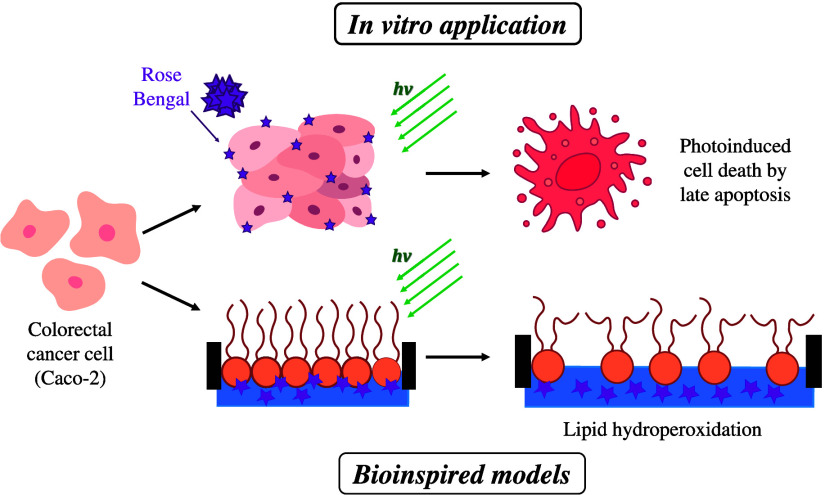

Rose Bengal (RB) is a promising photosensitizer (PS) for photodynamic therapy (PDT), but its application to colorectal carcinoma remains largely unexplored. Herein, we employ *in vitro* assays to demonstrate that incorporation of RB has substantial phototoxicity against Caco-2 cells, with more than 80% reduction in cell viability for 24 h incubation with 5 × 10^–6^ mol/L RB followed by irradiation. In contrast, RB had minimal toxicity without irradiation. The mechanisms of RB action were further elucidated using confocal fluorescence microscopy, Langmuir monolayers as cell membrane models, and flow cytometry to determine the cell death pathways. Flow cytometry revealed that the primary mode of cell death was late apoptosis. RB incorporation affected Caco-2 plasma membrane morphology under light irradiation, and membrane interactions were confirmed using Langmuir monolayers of Caco-2 lipid extracts. Incorporation of RB into the monolayers shifted the pressure–area isotherms toward larger molecular areas, especially at low surface pressures and increasing RB concentrations (1, 10, and 25 × 10^–6^ mol/L). RB adsorption also caused a decrease in the in-plane elasticity (Cs^1–^) of the Caco-2 monolayers, with a large increase in monolayer flexibility as RB concentration increased. According to polarization-modulated infrared reflection absorption spectroscopy (PM-IRRAS), the anionic RB interacted electrostatically with positively charged phospholipid groups. Moreover, the changes in surface area observed in the monolayers upon RB incorporation and irradiation could be attributed to hydroperoxidation reactions triggered by the generation of singlet oxygen (^1^O_2_). These findings indicate that RB may be used as a PS in the PDT of colorectal cancer, providing detailed insights into its mechanism of action and phototoxicity.

## Introduction

The World Health Organization (WHO) publishes annual data on global cancer incidence and mortality. According to the 2022 report, over 19 million cancer cases were recorded, resulting in more than 9 million deaths.^1^ Colorectal cancer ranks third in incidence and second in mortality, with over 900,000 deaths annually.^1,2^ The high mortality rate is largely attributed to late-stage diagnosis and the prevalence of metastasis, which complicates treatment and reduces the chances of cure.^3^ Current treatments primarily involve surgery, often in combination with chemotherapy and radiotherapy, both of which cause significant side effects.^4^ Given these challenges, there is an ongoing search for alternative therapies that offer more targeted and effective treatment while improving patients’ quality of life. In this context, Photodynamic therapy (PDT) is promising because it is minimally invasive,^5−9^ and can be applied across a wide range of treatments, including in antibiotic-resistant infections, superficial and deep cancers, dermatology, ophthalmology, cardiovascular, and neurological disorders.^10−16^

At the core of PDT is the administration of a photosensitizer (PS) to the target site, followed by exposure to light of a specific wavelength.^17,18^ This activation induces the PS to transition from its stable ground state (singlet state, ^1^PS) to an electronically excited singlet state (^1^PS*)^19,20^ that can dissipate energy via fluorescence or heat.^21^ It may also undergo intersystem crossing to reach a more stable, electronically excited triplet state (^3^PS*).^21^ This state is key for initiating type I or type II reactions, generating reactive oxygen species (ROS) that damage cellular components and induce cell death.^18^ Type I reactions generate free radicals through electron or hydrogen transfer to adjacent molecules, whereas type II reactions primarily involve the transfer of energy to oxygen molecules, producing singlet oxygen species (^1^O_2_).^22−24^ Although ^1^O_2_ are regarded as potent phototoxic agents in PDT, the short ^1^O_2_ lifetime (0.04 μs for biological systems and 4 μs for aqueous solutions) limits photodynamic damage to a nanometer-scale area (∼100 nm) surrounding them. Consequently, the efficacy of PDT depends on the positioning of the PS, which has prompted a search for PSs that interact specifically with the target, in addition to having a high quantum yield (ΦΔ) for ^1^O_2_.^19,20,25,26^

Among the PSs utilized in PDT,^27−29^ xanthene derivatives are noteworthy for their high ΦΔ ^1^O_2_ and strong affinity toward biological structures.^28,30−33^ Xanthene-based PSs include Rose Bengal (RB), Erythrosine B (EB), Eosin Y (EY), and Fluorescein (FL), which contain a common central chromophore with three condensed aromatic rings and an oxygen atom within the central ring.^33^ In particular, RB exhibits the highest ΦΔ^1^O_2_ (RB > EB > EY > FL) among the xanthene PSs, and is amphiphilic with a partition coefficient of 0.66.^34^ This amphiphilic property enhances RB integration into plasma membranes, potentially amplifying the photodynamic effects as unsaturated phospholipids are a primary target of ^1^O_2_. While the mechanisms governing photoinduced oxidation have been identified,^35^ the intricate molecular processes that result in biological damage are yet to be elucidated.^36^

The application of RB in the treatment of colorectal cancer was explored by Qin et al.,^37^ using xanthene as an immunomodulator in murine colon cancer. Additionally, Sztandera et al.^38^ investigated the RB interaction with colorectal cancer cells, identifying the OATP1B1/1B3 transporter as a key player in RB uptake, which influences its effectiveness in PDT. However, the molecular mechanisms behind the photooxidative processes triggered by RB irradiation remain unclear. Herein, we shall demonstrate the effectiveness of RB in PDT for cancer using *in vitro* systems.^40^ To elucidate the mechanisms behind the effectiveness of RB, Langmuir monolayers were utilized to create a bioinspired model of the colorectal cancer (Caco-2) cell membrane. This approach was aimed at determining the molecular-level mechanisms of RB insertion and the subsequent effects triggered by irradiation. Lipid extracts isolated from Caco-2 cell cultures were assembled in Langmuir monolayers to mimic the tumor membranes. Evidence of the RB adsorption was provided by surface pressure isotherms, while the molecular-level interactions were investigated with polarization-modulation infrared reflection–absorption spectroscopy (PM-IRRAS). The morphology of the monolayers was also analyzed by Brewster angle (BAM) and atomic force (AFM) microscopies.

## Experimental Section

### Materials and Cells

Phosphate-buffered saline (PBS, ref P4417), chloroform (CHCl_3_, 99.0–99.4%, ref.32211) and Rose Bengal (RB, ref.330000) were acquired from Sigma-Aldrich and used without additional purification. PBS solutions were prepared by diluting 1 PBS tablet in 200 mL ultrapure water (resistivity of 18.2 MΩ·cm) obtained from a Milli-Q system (model Direct-Q 3UV). A stock solution of RB was prepared in PBS at 10 mmol/L. Colorectal cancer cells (Caco-2), acquired from Cell Bank of Rio de Janeiro (ref.0059, BCRJ), were cultured in T-75 flasks (ref.3290; Corning Glass Works, Corning, NY, USA) in Roswell Park Memorial Institute medium (RPMI 1640, ref.23400021, Gibco). The cell culture was supplemented with 10% (v/v) of fetal bovine serum (FBS; ref.0521-500, Cultilab, Campinas, Brazil) and 1% of antibiotics and antimycotics (ref.15240-062, ThermoFisher). The culture was maintained in an incubator at 37 °C and 5% of CO_2_ atmosphere. The cells were subcultured every 2 days and rinsed with 3 mL of PBS, followed by the addition of 1 mL of trypsin (for 4 min at 37 °C) to detach the cells from the flask. Half of the harvested cells were transferred to a new T-flask containing fresh culture media to maintain the cell culture. The remaining cells were used for the *in vitro* assays, being seeded in 96-well plates at a density of 5 × 10^4^ cells per well to reach ∼80% of confluency within 24 h. The cells were then subjected to incubation with different concentrations of RB ranging from 0.25 to 25 × 10^–6^ mol/L for different periods of time (0.5, 3, and 24 h). For the phototoxicity assays, the cells were incubated for the same periods (0.5, 3, and 24 h), after which they were subjected to light irradiation for 1 h. A controllable LED source (at 525 nm; Biolambda, São Paulo, Brazil) with 32.26 mW/cm^2^ of power providing 116 J/cm^2^ of light dose was employed for irradiation. This high intensity was selected to evaluate the maximum photooxidative potential of the RB.

### MTT Colorimetric Assays

Cell viability was assessed before (non-irradiated) and after photoactivation (irradiated) using a colorimetric assay based on MTT [3-(4,5–7 dimethylthiazol-2-yl)-2,5-diphenyltetrazolium bromide] (ref M2003, Sigma-Aldrich). After the incubation with RB and subsequent irradiation, the cells were cultured with F12 medium for 24 h before the 1 h incubation with 0.5 g/L of MTT. The formed formazan crystals were diluted in to 50 × 10^−6^ μL dimethyl sulfoxide (DMSO; ref D4540, Sigma-Aldrich) and the absorbance measurements were taken at 560 nm using a Multiskan FC Microplate Photometer. Cells incubated only in the culture medium were considered 100% viable and used as control (CC), while cells irradiated in absence of RB were considered as light control (LC). The death control (DC) was obtained by adding hydrogen peroxide (H_2_O_2_, 50 × 10^–6^ mol/L) to the cell culture. The experimental data were analyzed using the GraphPad Prism 9, with a statistical analysis of variance at one-unpaired multiple *t* test, according to Bonferroni test (*p* ≤ 0.05). All measurements and procedures were carried out at least in triplicate.

### Confocal Fluorescence Microscopy

The localization of RB within cells was determined using fluorescence confocal microscopy on a Nikon C2/C2si Eclipse Ti-E inverted microscope (Nikon, Kyoto, Japan), equipped with a 40x air objective (NA 0.9). DAPI (4,6-diamidino-2-phenylindole; Invitrogen, catalog number R37606) was employed for labeling the cell nucleus, while the cell membrane was stained using WGA (Wheat Germ Agglutinin) Alexa Fluor 488 conjugate (Invitrogen, catalog number W11261). Caco-2 cells (3 × 10^5^ cells/well) were seeded on glass coverslips in 24-well plates, and three RB concentrations (1, 2.5, and 10 × 10^–6^ mol/L) were incubated for 3 h. Following LED irradiation (at 525 nm) using the BioLambda system (32.26 mW/cm^2^; São Paulo, Brazil), the medium containing RB was replaced with F12 medium, and the cells were incubated for 24 h. Subsequently, the supernatant was replaced by 300 × 10^–6^ L WGA Alexa Fluor (10 × 10^–6^ g/mL) and incubated for 10 min. Finally, the cells were rinsed with PBS and the glass coverslips were flipped over glass slides containing 10 × 10^–6^ L DAPI. The slides were sealed with colorless base nail (Risqué) and stored at 5–10 °C for further analysis. The RB fluorescence was used to identify the cellular region where the PS was incorporated. The microscope was configurated with the following light conditions: DAPI (excitation at 358 nm/emission at 455 nm), WGA (excitation at 488 nm/emission at 520 nm), and RB (excitation at 530 nm/emission at 620 nm).

### Flow Cytometry

Caco-2 cells (5 × 10^5^ cells/well) were treated with 0.25, 1, and 10 × 10^–6^ mol/L RB for 3 h, followed by irradiation according to the protocol in Section “Confocal Fluorescence Microscopy”. After irradiation, the cells were trypsinized (5 min at 37 °C), transferred to centrifuge tubes, and incubated with 2.5 × 10^–6^ L Annexin V-Alexa Fluor 488 and 1 × 10^–6^ L propidium iodide (PI; 10^–6^ g/L). Given the potential fluorescence overlap between RB (emission ∼574 nm) and PI (emission ∼615 nm), an RB-only control was included to assess any signal interference. This control ensured that RB fluorescence did not compromise data analysis. Moreover, the fluorescence detected from RB was used to evaluate its incorporation into the cells. Flow cytometry was performed using an Accuri C6 Plus (Becton Dickinson), and data were analyzed with BD Accuri C6 Plus software (Becton Dickinson).

### Lipid Extract from Caco-2 Cells

The lipid extract from Caco-2 cells was obtained by adapting earlier protocols.^39,40^ The Caco-2 cell culture was detached from the T-flask and transferred to a falcon tube. Following centrifugation with a Rotina 380 R (Tuttlingen, Germany) at 1500 rpm for 5 min, the supernatant was removed, and the cell pellet remained at the bottom of the falcon tube. Subsequently, 1 mL ultrapure water was added, and the mixture was vortex-stirred for 10 min. Then, 4 mL chloroform was introduced, followed by an additional 10 min of stirring. This solution was sonicated for 30 min and followed by centrifugation at 1500 rpm for 10 min, resulting in three distinct phases: the upper phase containing cell fragments soluble in ultrapure water, a middle fraction with the cell mass, and a lower phase with fragments soluble in chloroform.^39,40^ The chloroform-based solution, enriched with lipids from the Caco-2 cells, was transferred to a new amber flask.

### Langmuir Films of Caco-2 Lipid Extract

Langmuir films were prepared in a Langmuir trough (KSV-NIMA/KN 2002) by spreading a solution of Caco-2 lipid extract on PBS or PBS containing RB at 1, 10, and 25 × 10^–6^ mol/L. The spread lipid extract was left undisturbed for 10 min to ensure complete evaporation of the chloroform. The remaining material at the air–aqueous interface was then subjected to symmetrical compression using mobile barriers, with a compression rate of 3.75 cm^2^/min. Surface pressure (π) versus surface area isotherms were obtained with the Wilhelmy method,^41^ using a platinum rod. Note that the area is presented as the area per mL of extract rather than the traditional molecular area as the exact composition of the extract is not known. The subphase temperature was maintained at 21 °C with a thermostatic bath (SSDu-10L, SolidSteel, Piracicaba, Brazil). Although unsaturated lipids exposed to air can result in changes in the surface pressure due to lipid oxidation,^42^ no precautions were taken here to prevent oxidation. Nevertheless, the isotherms were reproducible, with surface pressure variations within 2 mN/m. The compressibility modulus of the monolayers (C_S_^–1^) was determined from the π–*A* isotherms applying the eq 1:
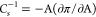
1where π is the surface pressure and A is the area.^39,40^

The effects of RB on Caco-2 monolayer stability were investigated at a fixed surface pressure of 30 mN/m, while monitoring the surface area over 2-h observation. The choice of this surface pressure was based on the literature, according to which the lateral pressure in the plasma membranes of eukaryotic cells is around 30 mN/m.^43^ Stability tests were conducted on Caco-2 lipid extract monolayers on PBS or RB subphases. Similarly, tests were conducted on Caco-2 lipid extract monolayers on photoactivated RB subphases, where irradiation commenced once the monolayer reached a pressure of 30 mN/m. For this, a LED irradiation system (at 525 nm) from BioLambda (32.26 mW/cm^2^; São Paulo, Brazil) was employed, positioned 20 cm above the Langmuir trough with an angle of 30°. The dynamic behavior of the system was characterized by monitoring time-dependent changes in the average relative area.

Polarization-modulated infrared reflection–absorption spectroscopy (PM-IRRAS, KSV PMI550) measurements were performed with an incidence angle of 81° and a resolution of 8 cm^–1^ to evaluate the molecular interactions between Caco-2 lipid extract monolayers and RB. The PM-IRRAS spectra were obtained from Caco-2 lipid extract monolayers on PBS and PBS containing RB (25 × 10^–6^ mol/L), at a constant surface pressure of 30 mN/m. For irradiated monolayers, PM-IRRAS spectra were promptly acquired following the 20 min irradiation period, performed with a LED source (50 W of power). Spectral reproducibility was ensured by conducting at least three experiments for each condition. Therefore, modifications in band position and/or relative intensity did not stem from spectral dispersion, but rather from interactions between the Caco-2 lipid extract monolayer and RB. Images from a Brewster angle microscope (BAM) were taken using an I-Elli2000 imaging ellipsometer (I-Elli2000, Nanofilm Technologies) coupled to a Langmuir trough (Nima Technology). This instrument is equipped with a 50 mW Nd:YAG laser (λ = 532 nm), and images were obtained using a 20× magnification lens with a lateral resolution of 1 μm and a 53.15° incident angle. The monolayers were compressed at a rate of 5 cm^2^ min^–1^. Atomic Force Microscopy (AFM) was used to image the Caco-2 lipid films in PBS and RB (25 × 10^–6^ mol/L). The films were transferred onto mica substrates (V1 quality, from Electron Microscopy Sciences) using the Langmuir–Blodgett (LB) deposition technique from the air/aqueous interface.^44^ In this method a freshly cleaved mica sheet was immersed into the subphase prior to lipid dispersion. Subsequently, the substrate was withdrawn from the subphase at a controlled rate of 3.75 cm^2^/min, keeping the surface pressure constant at 15 or 30 mN/m. A Bruker Multimode 8HR Nanoscope 9.7 (Digital Instruments, Santa Barbara, CA) was used to acquire the AFM images at the air–solid interface under ambient conditions. Images were captured using peak-force tapping mode at a scan rate of 0.3–1 Hz, employing a SCANASYST-AIR probe (with a silicon nitride cantilever, resonance frequency of 70 kHz, nominal spring constant of 0.4 N/m, and tip radius of 2 nm). Image analysis was conducted using Nanoscope software version 2.0.

## Results and Discussion

### Toxic and Phototoxic Effects of Rose Bengal on Caco-2 Cells

**Figure 1:**
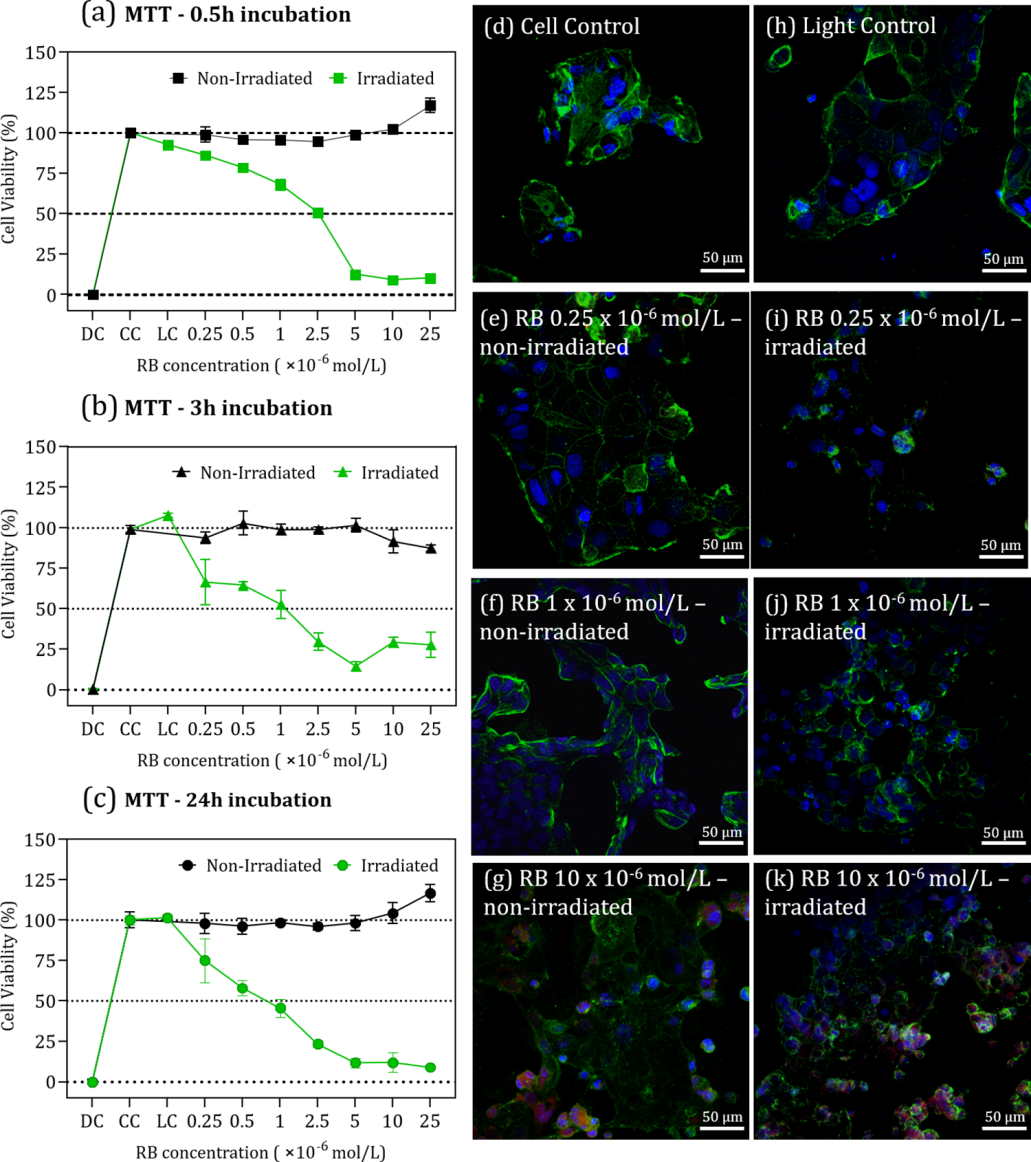
Toxic (non-irradiated, black line) and phototoxic (irradiated, green line) effects of RB in *in vitro* culture of Caco-2 cells, assessed by MTT assays. Cell viability was determined for cells incubated for (a) 0.5, (b) 3 and (c) 24 h with different concentrations of RB (from 0.25 to 25 × 10^–6^ mol/L). CC corresponds to cell control, DC to death control, and LC to light control. Confocal fluorescence microscopy of Caco-2 cells incubated for 3 h with 0, 0.25, 1, and 10 × 10^–6^ mol/L of RB in the dark (d to g, respectively) and after irradiation (h to k, respectively). The cell membrane (green) was stained with WGA Alexa Fluor 488 while the nucleus (blue) with DAPI. RB fluorescence was recorded at 620 nm (red).

Figure 1a–c shows the outcomes of MTT assays to assess both the toxicity and phototoxicity of RB at concentrations from 0.25 × 10^–6^ to 25 × 10^–6^ mol/L during three incubation intervals (0.5, 3, and 24 h). Exposure to RB without subsequent irradiation had no significant effect on cell viability, demonstrating the PS low toxicity across all incubation times. Cell viability was only significantly reduced with exposure to the 525 nm excitation light. The RB capacity to generate ROS, especially ^1^O_2_, underpins its phototoxic effects by initiating oxidative reactions within cells, leading to cell death. For all incubation times, cell viability decreased with increasing RB concentrations up to 5 × 10^–6^ mol/L, after which cell viability stabilized near 10%. For the 0.5-h incubation period, the concentration of RB required to reduce cell viability by 50% (CC_50_) was 2.26 × 10^–6^ mol/L (Figure S1a). This CC_50_ concentration diminished further to 0.68 × 10^–6^ and 0.63 × 10^–6^ mol/L for incubation periods of 3 and 24 h, respectively. These findings, depicted in Figure S1b,c, suggest the phototherapeutic impact depends on the exposure time to RB.

The correlation between the phototoxic effects of RB and its concentration over different incubation times is likely related to the extent of PS incorporation into cells. For example, Sztandera et al.^38^ observed a lower reduction in cell viability with RB incorporation in Caco-2 cells. Using 5 × 10^–6^ mol/L RB, a 5-h incubation, and 30 min of irradiation, they observed a 50% decrease in cell viability. Moreover, the efficacy of RB appears to differ across cell lines, as indicated in Table S1. Prior research with EB has shown that prolonged exposure results in reduced CC_50_ values, indicating enhanced incorporation of the PS over time.^45^ In the study by McEwan et al.,^46^ RB reduced murine melanoma cell viability by ca. 20% and 30% after 3 h of incubation and irradiation with white light doses of 11.4 and 22.8 J/cm^2^, respectively. Dhillon et al.^47^ observed similar effects across various cancer cell lines (Table S1) with RB, showing up to 40% viability reduction. Uppal et al.^48^ reported a 50 ± 5% and 15 ± 10% decrease in cell viability for oral and breast cancer cells, respectively, using RB. These studies indicate the variability in RB effectiveness for PDT, as a function of cell type, which can be due to different incorporation mechanisms and/or experimental setup.

The confocal microscopy images in Figure 1d-k were taken after a 3-h incubation with RB concentrations of 0.25, 1, and 10 × 10^–6^ mol/L. In the non-irradiated group (Figure 1d-g), cell morphology appeared consistent across all RB concentrations, as expected from the lack of toxicity within this range. At the highest concentration of RB (10 × 10^–6^ mol/L), fluorescence from the PS was detected around the cell membranes, thus pointing to a possible role of RB-membrane interactions in cellular uptake mechanisms. Similar research by Bistaffa et al.^39,45^ on HEp-2 and MCF-7 cells found no significant morphological changes in non-irradiated samples, with EB detection at concentrations starting from 10 × 10^–6^ mol/L primarily localized around the membrane areas. Large morphological changes were observed for irradiated samples in Figure 1h-k, which increased with RB concentration. Even at the lowest concentration of 0.25 × 10^–6^ mol/L, changes in membrane staining were evident, a pattern that was consistent at higher concentrations. At 10 × 10^–6^ mol/L, the RB fluorescence mirrored that of the non-irradiated samples. However, the RB distribution appeared more widespread in the irradiated group, suggesting a potential permeation and internalization of the PS within the cells. The nuclear size decreased for all RB concentrations, as indicated in Figure S2. Such morphological modifications suggest apoptotic and necrotic pathways, likely induced by oxidative stress from irradiation.^49^ The reduction in nuclear size is typical of apoptosis, as in cytoplasmic compaction, chromatin condensation, and DNA fragmentation.^50,51^

**Figure 2:**
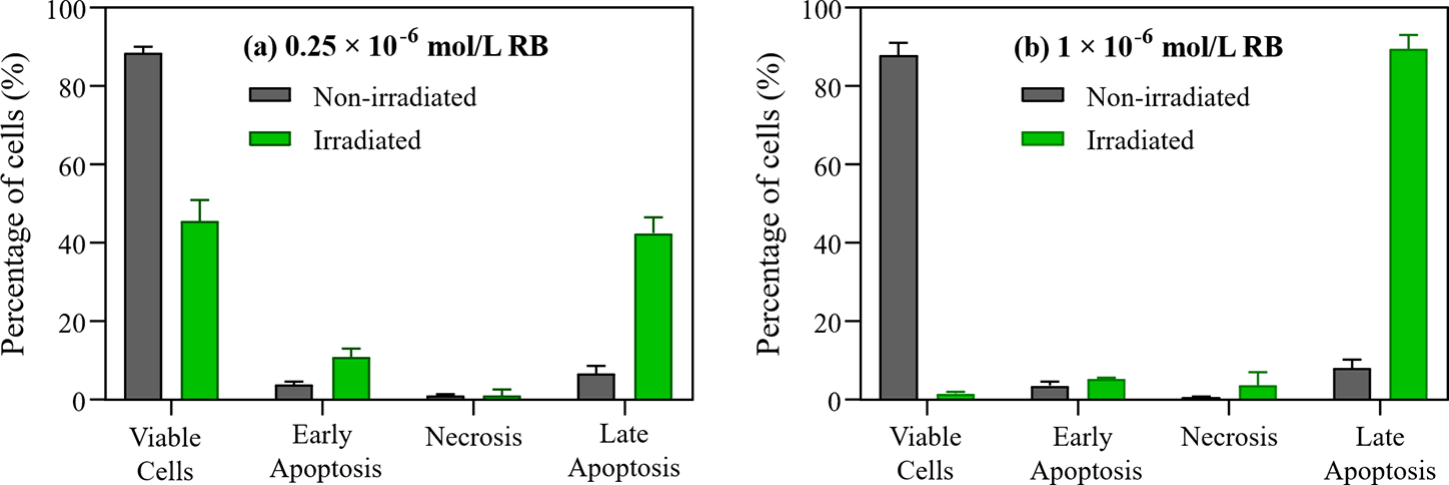
Percentage of Caco-2 cells undergoing apoptosis and necrosis based on the annexin V/PI flow cytometry assay for (a) 0.25 × 10^–6^ mol/L and (b) 1 × 10^–6^ mol/L RB after 3 h of incubation, both before and after irradiation. Corresponding cellular controls are provided in Figure S3 (Supporting Information).

To determine the activated pathways, apoptosis and necrosis were evaluated using the annexin V/propidium iodide (PI) double-staining protocol after 3 h of incubation with 0.25 and 1 × 10^–6^ mol/L RB (Figure 2a,b). RB data at 10 × 10^–6^ mol/L is compromised by its significantly high fluorescence and is not shown. In apoptotic cells, phosphatidylserines translocate to the outer membrane, allowing annexin V binding, while necrotic cells expose nuclear material, which binds PI. Viable cells are identified by the absence of fluorescence (double-negative), early apoptotic cells by annexin V-positive/PI-negative staining, necrotic cells by PI-positive/annexin V-negative staining, and late apoptotic cells by double-positive fluorescence.^52,53^ In the 0.25 × 10^–6^ mol/L RB group, 42 ± 4% of the irradiated cells were in late apoptosis. This percentage increased significantly to 89 ± 4% when RB concentration was raised to 1 × 10^–6^ mol/L. The rise in late apoptotic cells correlates with higher RB incorporation, as suggested by confocal microscopy (Figure 1d–k). Despite the cell viability data from the MTT assay (Figure 1b), the apoptotic cascade was clearly activated after 3 h of incubation with 1 × 10^–6^ mol/L RB following irradiation, suggesting that the efficacy of RB-mediated PDT may be greater than indicated by the MTT results.

The results above point to an important role of the modifications in the plasma membrane for PDT. However, they are not sufficient to determine the molecular-level interactions responsible for the membrane effects in PDT. This can be achieved using cell membrane models, as it was done here with Langmuir monolayers^46^ to be discussed next.

### Langmuir Monolayers as Caco-2 Cell Membrane Models

**Figure 3:**
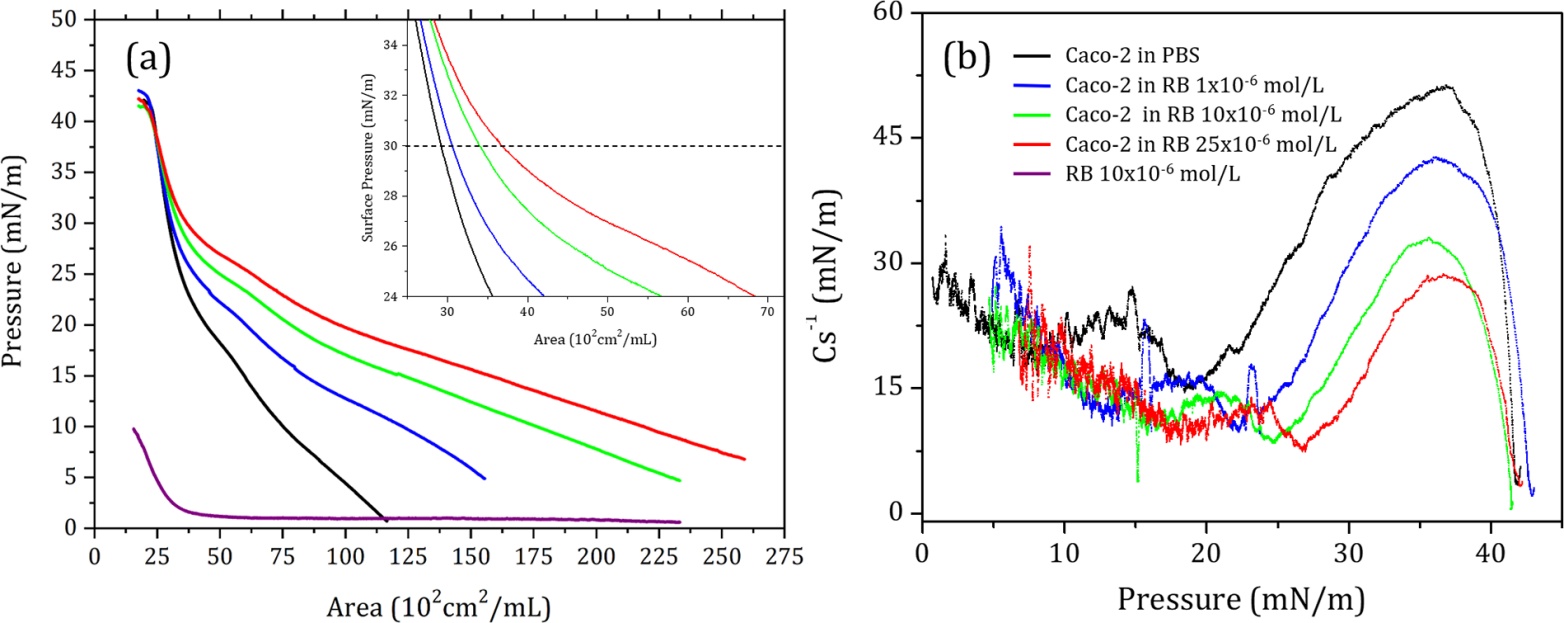
(a) Surface pressure (mN/m) *versus* area (cm^2^/mL of lipid extract) isotherms of Caco-2 lipid extract on PBS and RB (1, 10, and 25 × 10^–6^ mol/L), and pure RB (at 10 × 10^–6^ mol/L). The inset provides a close-up view of the isotherm curves at 30 mN/m, from which the displacements in the relative area were measured. (b) C_S_^–1^ of the Caco-2 lipid extract on PBS and RB solution. C_S_^–1^ was calculated from the π–*A* isotherms using the eq 1.

The plasma membrane of Caco-2 cells can be simulated using Langmuir monolayers from Caco-2 lipid extract. The surface pressure (π) *versus* area (cm^2^/mL) isotherms of this extract on PBS and different RB concentrations are shown in Figure 3a. RB in PBS was surface active, but could not form stable monolayers. RB incorporation into Caco-2 films shifted the isotherms toward larger areas as the subphase concentration of RB was increased, especially at low surface pressures. At higher surface pressures, the isotherms converged, suggesting that some RB molecules adsorbed on the monolayers were expelled into the subphase. Table 1 lists the relative area shifts when comparing the extrapolated the area from 30 mN/m (inset of Figure 3a) to 0 mN/m of the RB curves (*A*_*RB*_) with the PBS curve (*A*_*PBS*_). The relative area shift was obtained using eq 2:
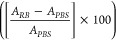
2where *A*_*PBS*_ and *A*_*RB*_ are the extrapolated areas at 30 mN/m for Caco-2 lipid extract on PBS and RB solutions, respectively. The 30 mN/m surface pressure is regarded as representative of the lateral pressure in cell membranes.^54^ Previous research on anionic xanthene-derived PSs revealed a stronger interaction with cationic lipid groups, attributed to electrostatic attractions. This resulted in higher adsorption rates with zwitterionic phosphatidylcholine (PC)^45,55^ monolayers and lower with anionic phosphatidylserines.^45^ In healthy mammalian cells, the outer leaflet of plasma membrane is predominantly composed of PC. Other lipids such as phosphatidylserine and phosphatidylethanolamine are predominantly confined to the inner leaflet, without being expressed significantly in the outer leaflet.^56,57^ Such lipid asymmetry is absent in tumor cells.^58^ Despite the varying complexity in lipid structures, it may be presumed that the primary mechanism facilitating insertion of the anionic RB into the Caco-2 extract monolayer is the electrostatic attraction with positively charged moieties of the phospholipids.^58^ As the RB concentration in the subphase increased, the C_s_^–1^ for Caco-2 monolayers decreased (Figure 3b), indicating an increased flexibility of the films. The capability to modulate cell membrane elasticity facilitates mass transport across cell membranes by reducing their selectivity, thereby increasing the absorption of PS molecules within the lipid film.^59^ Comparable outcomes were noted with the use of different photosensitizers, including EB, eosin decyl ester (EosDec), toluidine blue, and nanostructures such as gold shell-isolated nanoparticles (AuSHINs).^39,55,60−62^

**Table 1:** Relative area shifts per cell lipid extract on different concentrations of RB

RB concentrations (mol/L)	Relative area shifts on Caco-2 cell extract (%)
1 × 10^–6^	22 ± 2
10 × 10^–6^	60 ± 1
25 × 10^–6^	140 ± 6

**Figure 4:**
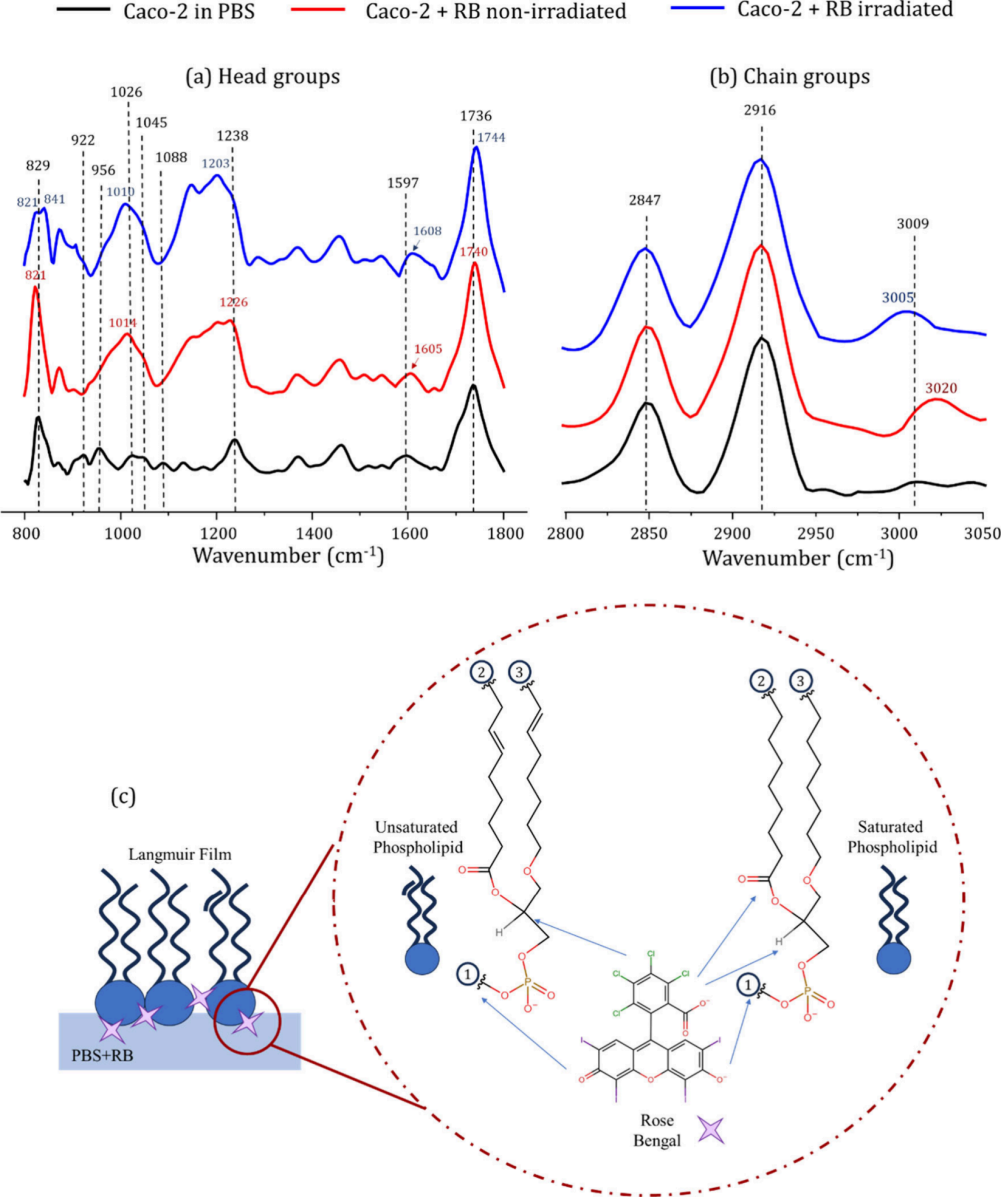
PM-IRRAS spectra of Caco-2 lipid extract monolayers on PBS and RB (25 × 10^–6^ mol/L), before and after irradiation. The vibrational modes of the head groups are displayed in (a), whereas those of the chain groups are presented in (b). Scheme of the proposed interaction between Caco-2 lipid extract and Rose Bengal (RB) based on the PM-IRRAS data (c). The lipid extract contains different phospholipids with different chain sizes (represented by number 2 and 3). The RB molecule interacts mainly with the head groups of the phospholipids, both the phosphates, and cationic groups, (e.g., choline, number 1).

The molecular-level mechanism of RB insertion into Caco-2 monolayers was assessed using PM-IRRAS, with experiments performed on PBS and RB solutions (25 × 10^–6^ mol/L). The spectra are shown in Figure 4, while the band assignment is given in Table 2. The lipid composition of the extract was not determined; however, the lipid extract from Caco-2 cells is known to contain predominantly phosphatidylcholine (PC), sphingomyelin, and phosphatidylethanolamine. It also includes considerable amounts of phosphatidylserine, glycosphingolipids, and phosphatidylinositol.^63,64^ RB incorporation had stronger effects on the polar groups of Caco-2 phospholipids (Figures 4a) than on the aliphatic region (Figure 4b). For instance, γ_r_(CH_2_) at 829 cm^–1^ shifted to 821 cm^–1^ and υ_as_ (PO_2_^–^) shifted from 1238 to 1226 cm^–1^, accompanied by an increase in intensity. These displacements indicate that RB incorporation might result in hydrogen-bonded PO_2_ in the films, suggesting hydration of the phosphate groups.^62^ Further changes induced by RB included the shift of υ_as_(COO^–^) from 1597 to 1605 cm^–1^ and the disappearance of υ_as_ (CN^+^(CH_3_)_3_) at 956 cm^–1^, the first related to the carboxylate groups of phosphatidylserines and the latter to the choline headgroups of PC.^40,62,65^ Therefore, it may be presumed that electrostatic interactions are a key factor in the adsorption of RB onto Caco-2 films, as previously hypothesized. Regarding the chain groups, the only discernible difference in the spectra was observed in the υ_as_ (HC = CH) mode, which shifted from 3009 to 3020 cm^–1^. There was no alteration in the ordering mode of the aliphatic chains, as indicated by the I υ_s_ (CH_2_) /I υ_as_(CH_2_) ratio, which remained relatively unchanged (0.57 ± 0.01 on PBS, and 0.60 ± 0.05 on RB non-irradiated). Such behavior differs from previous studies involving other xanthene derivatives like EB, EY, and EosDec.^39,55,66,67^ The latter derivatives had more substantial interactions with polar groups, which led to changes in the ordering of aliphatic tails. This distinct behavior of RB suggests that attractive electrostatic forces may confine RB adsorption to the cationic groups within the Caco-2 phospholipid heads at the interface (as illustrated in the scheme of Figure 4c).

**Table 2:** Assignments of the main bands for Caco-2 monolayers and shifts induced upon RB interaction and irradiation

Caco-2 lipid extract (cm^–1^)			
PBS	RB	RB + irradiation	Assignments
829	821	821 and 841	γ_r_(CH_2_)
922	-	-	δ(NH_3_^+^)_rocking_ + υ(C–N)
956	-	-	υ_asv_(CN^+^(CH3)_3_)
1026	1014	1010	υ_as_ (CNC)
1045	-	-	υ_as_ (C–O–PO_2_^–^)
1088	-	-	υ_s_ (PO_2_^–^)
1238	1226	1203	υ_as_ (PO_2_^–^)
1597	1605	1608	υ_as_ (COO^–^)
1736	1740	1744	υ (C = O)
2847	2847	2847	υ_s_ (CH_2_)
2916	2916	2916	υ_as_ (CH_2_)
3009	3020	3005	υ (HC = CH)

**Figure 5:**
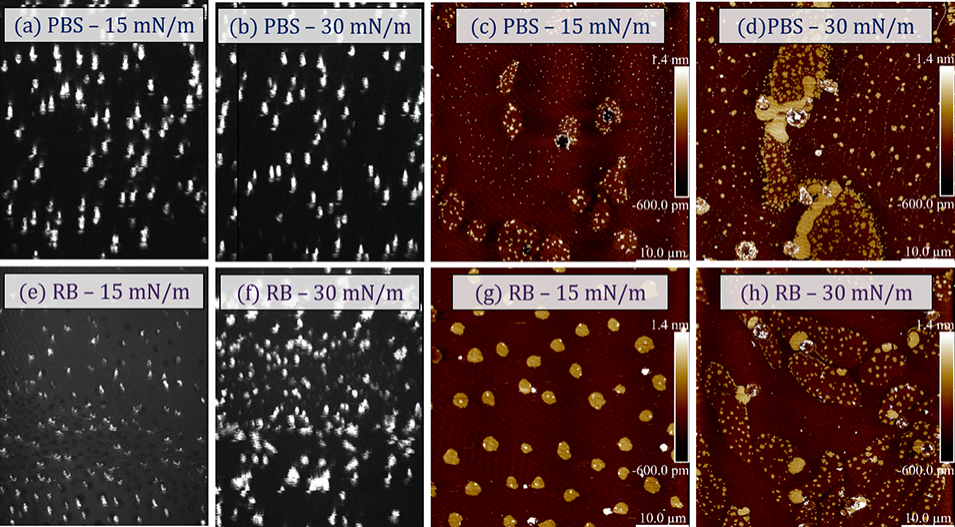
Brewster Angle Microscopy (BAM) images of Caco-2 monolayers on PBS (a-b) and on 25 × 10^–6^ mol/L of RB (e-f). AFM images of LB films deposited from Caco-2 on PBS monolayers (c-d) and Caco-2 on 25 × 10^–6^ mol/L of RB monolayers (g-h).

BAM images of Caco-2 monolayers on PBS and RB (25 × 10^–6^ mol/L) at surface pressures of 15 and 30 mN/m are displayed in Figure 5 (left panels). At 0 mN/m (result not shown), the surface morphology of both conditions (in the presence or absence of RB) is smooth owing to minimal intermolecular interactions. As the barriers closed and the monolayer surface pressure increased up to 30 mN/m, a phase characterized by circular bright spots emerged. These bright spots suggest the formation of thick aggregates in certain regions of the film, which are possibly responsible for the increase in film thickness observed in the data obtained through AFM, which will be discussed later. This behavior might relate to the initial structural arrangement of the Caco-2 phospholipids. At 15 mN/m, there is a noticeable influence on the size of the bright spots with RB, whereas at 30 mN/m the morphology closely resembles that seen in PBS, indicating a minimal impact on the organization of the monolayers at the micron scale.

The impact of RB incorporation into Caco-2 monolayers is more pronounced at the nanometer scale, as evidenced by AFM images recorded for LB films deposited at 15 and 30 mN/m from PBS (Figure 5c,d) and RB (Figure 5g,h) subphases. Table 3 lists the average domain height and diameter, and film roughness derived from the images analyzed. For an LB film deposited at 15 mN/m from a Caco-2 monolayer on PBS, elliptical domains appear alongside others that are more diffusely arranged. However, in examining the images more closely they are clearly multiple populations of domains. At 15 mN/m, there are larger elliptical domains that are <1 nm above the background matrix, smaller brighter domains atop the elliptical ones that at approximately 1.4–1.5 nm above the background, and few small aggregates that are much higher and quite variable in height leading to an average height of 4.4 nm (domain heights in Table 3). As for the RB-containing LB film, the average diameter of the brighter domains increased from 1.0 to 2.4 μm and the average height increased from 4.4 to 10.6 nm. These different populations remain at 30 mN/m, albeit with slight reductions in both height differences, likely due to the increasing thickness of the liquid-expanded matrix as it is compressed. At this biologically relevant surface pressure, there are no significant differences in the morphologies which suggests the incorporation of the RB itself into the membrane is not detrimental to its properties. Only the average domain height is affected by RB incorporation, increasing from 2.6 to 5.4 nm; both values are too high for a monolayer domain and suggest bi- or multilayered structures are formed. These findings align with the data obtained from surface pressure isotherms, sustaining that RB molecules have limited insertion into the closely packed monolayers (at high pressures), and tend to be primarily located underneath the monolayer. This is also in agreement with the isotherms that show that the RB is beginning to be squeezed out of the film at this surface pressure.

**Table 3:** Average height and diameter of bright domains and film roughness for the LB films produced with a Caco-2 monolayer on PBS, and with a Caco-2 monolayer on 25 × 10^–6^ mol/L RB^a^

	15 mN/m		30 mN/m	
Average	PBS	RB	PBS	RB
Height (nm)	4.4	10.6	2.6	5.4
Diameter (μm)	1.0	2.4	0.9	0.9
Roughness (nm)	0.2	0.2	0.3	0.3

a Data are shown for LB films fabricated at 15 and 30 mN/m, being obtained using Nanoscope 2.0 Particle Analysis (Figure S4).

### RB Photoactivation into Caco-2 Lipid Extract Monolayers

**Figure 6:**
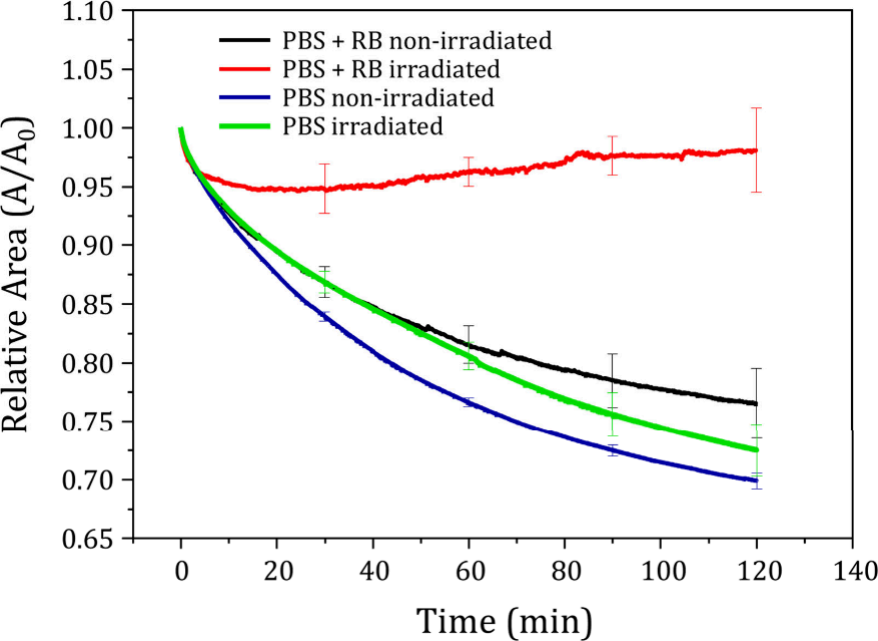
Relative area (A/A_0_) evolution of Caco-2 lipid extract monolayer on PBS non-irradiated (royal blue) and irradiated (green), and PBS + RB solution (25 × 10^–6^ mol/L) non-irradiated (black) and irradiated (red). A_0_ is the area at time *t* = 0, and A is the area at a given time t.

The impact of RB photoactivation on Caco-2 monolayers was investigated under a constant surface pressure of 30 mN/m over a 2-h period. Figure 6 shows the relative surface area (A/A_0_) evolution for monolayers on PBS, and both irradiated and non-irradiated monolayers on 25 × 10^–6^ mol/L RB. Subsidiary experiments indicated that irradiation of the monolayers on PBS led to no significant changes, as indicated in Figure 6.^39,61,62^ The decrease in relative area across all non-irradiated monolayers might be due to uncontrolled lipid oxidation from environmental ROS, resulting in the loss of material into the subphase.^68,69^ The incorporation of RB resulted in improved stability, evident from the smaller decrease in the relative surface area of monolayers containing 25 × 10^–6^ mol/L of RB (0.77 ± 0.03) over 120 min, compared to those on PBS (0.70 ± 0.01). Additionally, with RB present, the irradiated films showed relative surface areas higher than the non-irradiated, increasing to 0.95 ± 0.02, 0.96 ± 0.01, and 0.98 ± 0.04 after 30, 60, and 120 min, respectively. The difference in area between irradiated and non-irradiated films, reaching approximately 22% ± 7% after 120 min, surpasses the small 3.5% increase observed with EB in monolayers of MCF7 lipid extract.^39^ This difference could be attributed to RB enhanced efficiency in generating ROS which should affect the structure of the monolayers.^28^ Indeed, excited RB interacts with surrounding O_2_ producing singlet ^1^O_2_, a highly reactive specie capable to oxidize unsaturated lipid chains.^70^ The hyperoxide formation is the most likely outcome of the oxidative reactions, disrupting the membrane hydrophilic–hydrophobic balance and leading to conformational changes in the lipids that expanding the lipid molecular area for the monolayer.^71^

**Figure 7:**
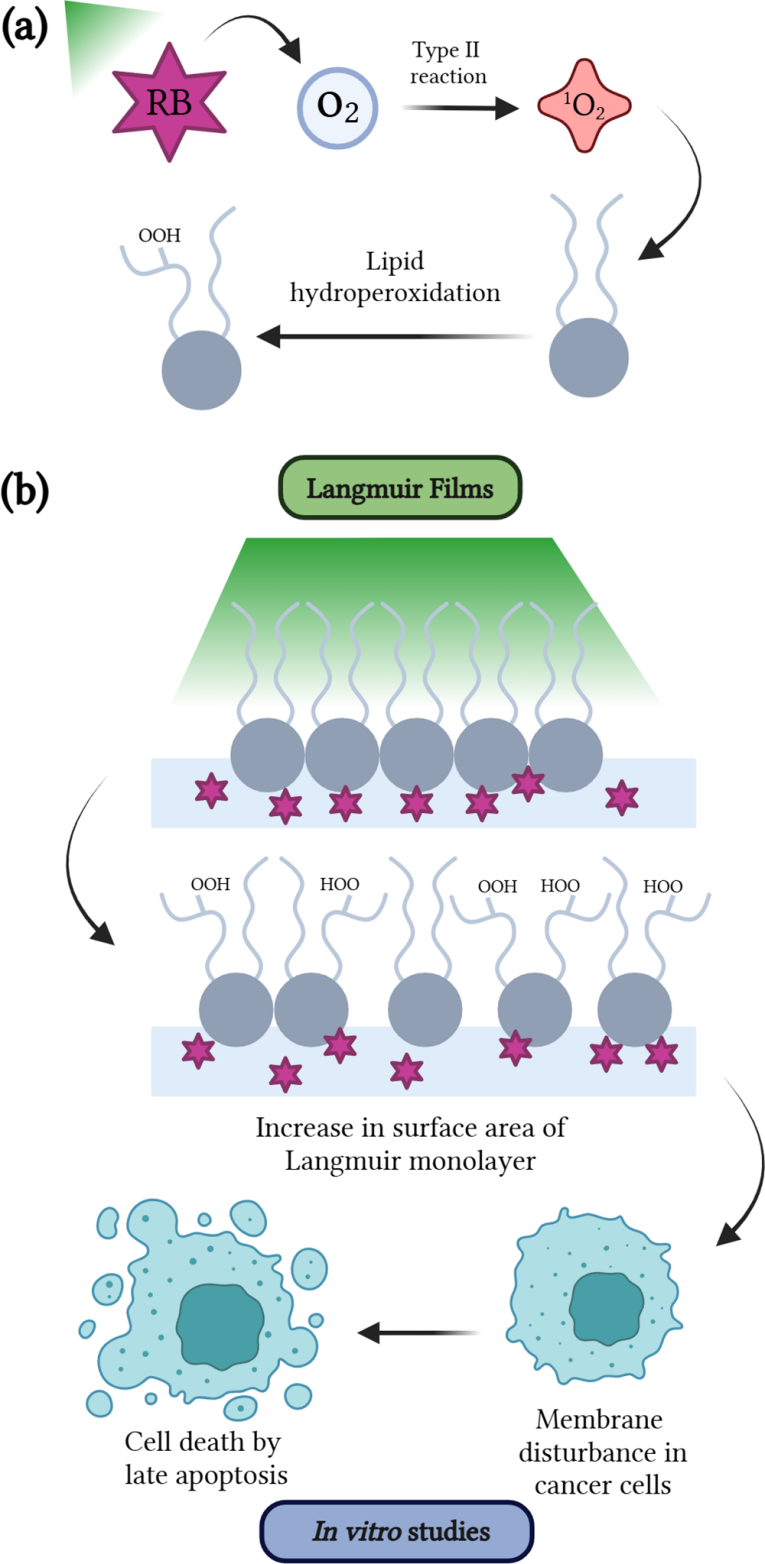
(a) Schematic representation of the oxidative processes triggered by the activation of the RB photosensitizer, producing ^1^O_2_ and leading to hydroperoxides in the lipids. (b) Photoinduced effects of RB on Langmuir films (hydroperoxide formation) and their correlation with the findings from *in vitro* assays. Created in BioRender. Ferreira, A. (2025) https://BioRender.com/a94g256.

Figure 4 shows that irradiation of Caco-2 monolayers in the presence of RB induces considerable modifications in the PM-IRRAS spectra for polar groups. For instance, γ_r_(CH_2_) at 821 cm^–1^ splits into two bands at 821 and 841 cm^–1^, accompanied by a decrease in relative intensity. The shift of υ_as_(PO_2_^–^) from 1226 to 1203 cm^–1^ suggests modifications in the hydrogen bonding between phosphate groups and nearby water molecules,^72^ which is consistent with the presence of hydroperoxides at the monolayer–subphase interface.^55^ In the nonpolar region, υ_s_(CH_2_) and υ_as_(CH_2_) were not affected during photoactivation. The I υ_s_(CH_2_)/I υ_as_(CH_2_) ratio shifted slightly from 0.60 ± 0.05 to 0.55 ± 0.05, suggesting that RB photoactivation does not impact the conformation of the aliphatic tails.^73^ A similar conclusion was reached for EosDec, of the same family of RB. EosDec had minimal impact on aliphatic groups in irradiated DOPE monolayers, but slightly reduced chain disorder in DOPG monolayers.^67^ Moreover, while the exact modification of the HC = CH vibrational mode cannot always be predicted precisely, and may vary across different systems, the shift from 3020 to 3005 cm^–1^ in the irradiated film is consistent with the generation of hydroperoxides at the unsaturation site.^67^ It is known that hydroperoxides may undergo cleavage to yield ketones, aldehydes, or carboxylic acids.^74^ Therefore, one may wonder if the (HC = CH) band in the irradiated spectrum indicates lipid chain breakage and membrane permeabilization. However, since RB is known as a type II PS it is likely that mostly hydroperoxidized lipids are present in the monolayer to be probed.^74,75^Figure 7 provides an overview of the main findings. Panel 7a illustrates how RB photoactivation generates ^1^O_2_, which oxidizes phospholipids to form hydroperoxides, representing the primary oxidation mechanism in the films. Panel 7b highlights the increase in surface area observed in the lipid monolayers, supported by PM-IRRAS data. These effects are further corroborated by i*n vitro* results, where hydroperoxide formation disrupts membrane organization, ultimately leading to cell death by late apoptosis.

## Conclusions

This study was aimed at elucidating the mechanisms through which Rose Bengal (RB) acts as a photosensitizer (PS) in photodynamic therapy (PDT) targeting colorectal cancer. MTT assays indicated minimal toxicity against Caco-2 cell lines from RB without irradiation, even at high concentrations and prolonged exposure. However, with light irradiation there was a significant reduction in cell viability, over 80% at concentrations above 5 × 10^–6^ mol/L. Confocal microscopy revealed changes in cellular and nuclear morphology indicative of late apoptosis, with effective RB uptake at 10 × 10^–6^ mol/L, which were consistent with the flow cytometry data. The importance of RB action on cell membranes was confirmed by simulating Caco-2 membrane using Langmuir monolayers made with a Caco-2 lipid extract. RB was incorporated into the lipid extract monolayers, indicated by a π–*A* isotherm shift of up to 140% at a concentration of 25 × 10^–6^ mol/L. Polarization-modulated infrared reflection–absorption spectroscopy (PM-IRRAS) provided evidence of RB interaction with cell membranes, driven by electrostatic interaction with phosphate, carboxylate, and choline groups of phospholipids. At the micron scale, Brewster angle microscopy (BAM) showed minimal changes in morphology upon RB incorporation. Detailed changes in monolayer structure determined by atomic force microscopy (AFM), showed an increase in the average domain height from 2.6 nm in PBS to 5.4 nm in RB, but otherwise similar morphologies at biologically relevant pressures. This highlights that it is only the irradiation, not the incorporation of RB, that causes cell membrane damage, in agreement with the toxicity assays. Stability tests indicated that RB irradiation leads to hydroperoxidation reactions, showing a significant 21.6% ± 6.5% area difference between irradiated and non-irradiated monolayers. In summary, the mechanisms of action of RB could be correlated with its interaction with Caco-2 cell membranes, while also confirming its potential as a PS in PDT for treating colorectal cancer.

## Supporting Information Available

List of studies that applied PS RB in PDT; Curves of CC_50_ in each incubation period; Bar graphs showing the variation of nucleus diameter at different RB concentrations with and without irradiation; Bar graph of flow cytometry of cell control; Images of AFM showing the areas analyzed by the software (PDF)
