# Supplementary material for: Photoactivated Rose Bengal Triggers Phospholipid Hydroperoxidation and Late Apoptosis in Colorectal Cancer Cells
Source: Langmuir. 2025 Mar 6;41(10):6789–800. doi: 10.1021/acs.langmuir.4c05013 (PMC11924238; doi:10.1021/acs.langmuir.4c05013)
Supplement: Supplementary file 1 — la4c05013_si_001.pdf [file la4c05013_si_001.pdf]

Supporting Information

Photoactivated Rose Bengal Triggers Phospholipid Hydroperoxidation and Late Apoptosis in Colorectal Cancer Cells

André Satoshi Ferreira [a]; Alexandre Mendes de Almeida Junior [a]; Mirella Boaro Kobal [a]; Lucas Gontijo Moreira [a]; Sabrina Aléssio Camacho [a]; Karina Alves de Toledo [a]; Osvaldo N. Oliveira Jr. [b]; Christine E. DeWolf [c]; Pedro Henrique Benites Aoki [a*].

[a] São Paulo State University (UNESP), School of Sciences, Humanities and Languages, Assis, SP, 19806-900, Brazil
[b] University of Sao Paulo (USP), São Carlos Institute of Physics, São Carlos, SP, 13566-590, Brazil
[c] Concordia University, Department of Chemistry and Biochemistry and Centre for NanoScience Research, Montreal, QC, H4B 1R6, Canada

*pedro.aoki@unesp.br

Table of Contents

List of Tables

Table S1. List of studies that applied PS RB in PDT using different conditions and cell lines..S2

List of Figures

Figure S1. CC_{50} of RB incubated for (a) 0.5, (b) 3 and (c) 24 h followed by irradiation. The data were obtained through a logarithmic regression using the data in Figure 1a-c S3
Figure S2. Mean values of cell nuclei diameter before and after irradiation of Caco-2 cells. Approximately 40 cells were counted for each group using ImageJ®. * $p < 0.05$ (multiple t test, Bonferroni), in relation to the non-irradiated population..S4
Figure S3. Cell death pathways for Caco-2 when no RB is added. The cells were incubated for 3h with F12 medium, followed or not by irradiation. S4
Figure S4. Treated AFM images of Caco-2 monolayers on PBS and RB at 15 mN/m (a and c) and 30 mN/m (b and d): the areas in cyan are the parts that were analyzed by the software. The roughness was obtained considering the whole image S5

Table S1. List of studies that applied PS RB in PDT using different conditions and cell lines.

Study	Cell lineage	Incubation time	Light Source	Light Dose (J/cm^2)	Viability reduction
This study	Caco-2	0.5, 3 and 24 h	525 nm LED source	116	Approximately 91% with 24 h incubation time
McEwan *et al.*	B16-F10-Luc2	3 h	White light	11.4 and 22.8	Approximately 30% with 22.8 J/cm^2
Dhillon *et al.*	B16-F10-Luc2, MCF-7, Panc-01, and HeLa	3 h	White light	22.8	Approximately 30% for B16-F10-Luc2, 20% for MCF-7, 35% for Panc-01, and 40% for HeLa cells
Uppal *et al.*	4451 and MCF-7	4 h	White light	Non-defined	Approximately 50% for 4451, and 15% for MCF-7 cells
Sztandera *et al.*	Caco-2, HT-29, HepG2, and Hep3B	5h	Q. Light Pro Unit lamp	72	IC50 of 19.84, 20.64, 5.03, and 2.75 for Caco-2, HT-29, HepG2, and Hep3B, respectively

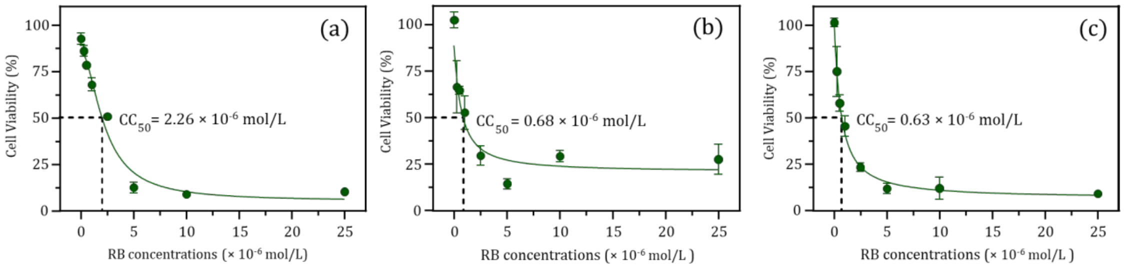

Figure S1. CC_{50} of RB incubated for (a) 0.5, (b) 3 and (c) 24 h followed by irradiation. The data were obtained through a logarithmic regression using the data in Figure 1a-c.

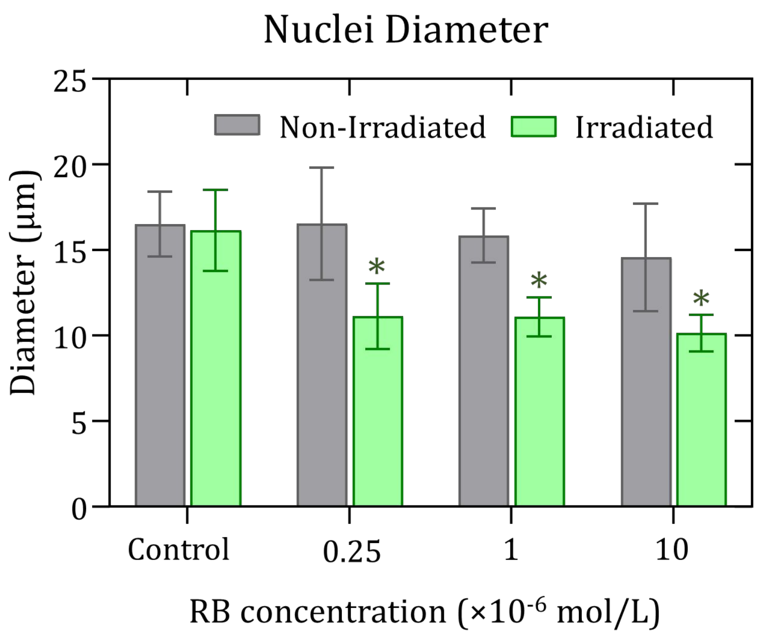

Figure S2. Mean values of cell nuclei diameter before and after irradiation of Caco-2 cells. Approximately 40 cells were counted for each group using ImageJ®. * $p < 0.05$ (multiple t test, Bonferroni), in relation to the non-irradiated population.

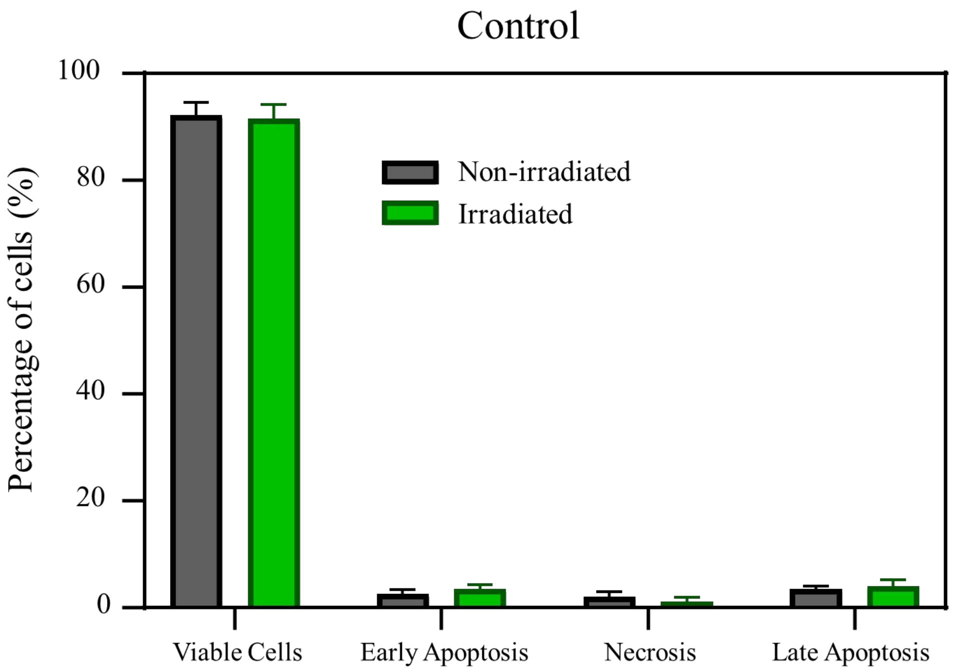

Figure S3. Cell death pathways for Caco-2 when no RB is added. The cells were incubated for 3h with F12 medium, followed or not by irradiation.

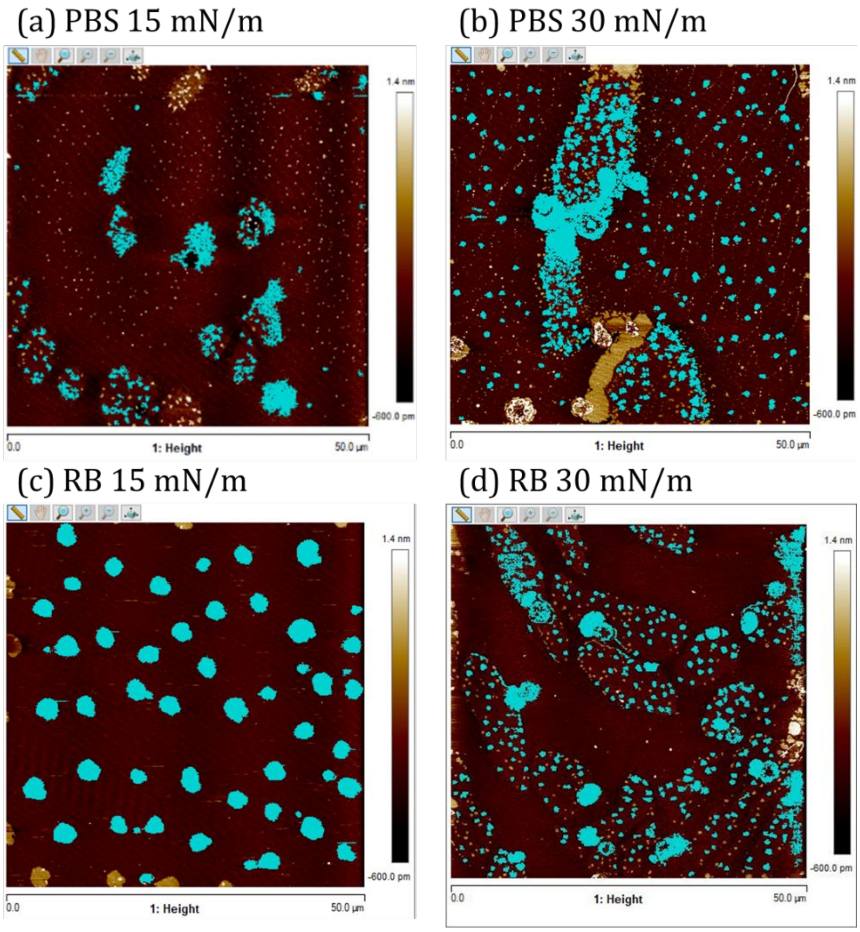

Figure S4. Treated AFM images of Caco-2 monolayers on PBS and RB at 15 mN/m (a and c) and 30 mN/m (b and d): the areas in cyan are the parts that were analyzed by the software. The roughness was obtained considering the whole image.
